# How to Deal with Median Arcuate Ligament during Pancreaticoduodenectomy? Management Strategies and Impact on Perioperative Outcomes

**DOI:** 10.1245/s10434-026-19830-8

**Published:** 2026-05-23

**Authors:** Elena Fernandez-de-Sevilla, Maxime K. Collard, Raffaele Vincenzo de la Rosa, Sophie Laroche, Iolanda Scoleri, Mehdi Boubaddi, Charles de Ponthaud, Julie Perinel, Julie Leclerc, Ruth Bustamante, Pietro Addeo, Rim Cherif, Ahmet Ayav, Mustapha Adham, Sebastien Gaujoux, Christophe Laurent, Stephanie Truant, Antonio Sa Cunha, Laurent Sulpice, Maximiliano Gelli, French Association of Hepato-Bilio-Pancreatic Surgery and Liver Transplantation (ACHBPT)

**Affiliations:** 1 Department of Anaesthesia, Surgery and Interventional Radiology, Gustave Roussy Hospital, University of Paris-Saclay, Villejuif, France; 2 Department of Hepatobiliary and Digestive Surgery, Rennes University Hospital, Rennes, France; 3 Department of Hepatobiliary Surgery and Transplantation, AP-HP Hôpital Paul-Brousse, University of Paris-Saclay, Villejuif, France; 4 Department of Digestive Surgery and Transplantation, Lille University Hospital, Lille, France; 5 Department of Digestive Surgery, Centre Magellan–CHU Bordeaux, Bordeaux, France; 6 Department of Hepato-Biliary and Pancreatic Surgery and Liver Transplantation, AP-HP, Pitié-Salpêtrière Hospital, Paris, France; 7 Department of Digestive Surgery, Edouard Herriot Hospital, Hospices Civils de Lyon, Lyon, France; 8 Department of Surgery, Nancy University Hospital Lorraine and Lorraine University, Vandoeuvre-lès Nancy, France; 9 Department of Digestive and Hepato-Pancreatic-Biliary Surgery, Hôpital Henri-Mondor, AP-HP, Paris Est Créteil University, UPEC, Créteil, France; 10 Hepato-Pancreato-Biliary Surgery and Liver Transplantation, Pôle des Pathologies Digestives et Hépatiques, Hôpital de Hautepierre-Hôpitaux Universitaires de Strasbourg, Université de Strasbourg, Strasbourg, France

**Keywords:** Median arcuate ligament, Celiac artery stenosis, Pancreaticoduodenectomy, Postoperative ischemia, Multicenter study

## Abstract

**Background:**

Median arcuate ligament (MAL) can cause coeliac trunk (CT) stenosis, which is often asymptomatic but may lead to severe hepatic, splenic, or digestive ischemia following pancreaticoduodenectomy (PD). Optimal diagnosis and management remain debated. The purpose of this study was to evaluate management strategies and perioperative outcomes of patients with MAL undergoing PD.

**Patients and methods:**

A retrospective multicenter study across ten high-volume French centers included patients diagnosed with MAL (hemodynamically significant or not) who underwent PD between 2005 and 2024. Diagnosis and management strategies and postoperative outcomes were collected.

**Results:**

MAL stenosis was suspected in 1.3% of patients (115/8676), with 59 cases (0.7%) showing hemodynamically significant MAL. MAL stenosis was suspected before surgery in 90% of patients (*n* = 104). Overall, 8 patients (13.8%) underwent preoperative stenting, 48 patients (82.8%) underwent intraoperative MAL division, and 2 patients (3.4%) underwent primary vascular reconstruction. Seven failures of MAL division (14%) required vascular reconstruction. Overall morbidity (Clavien–Dindo ≥ III) was 25%, pancreatic fistula B–C was 10%, biliary fistula was 6.9%, hemorrhage was 15%, and mortality was 4.3% (*n* = 5). Ischemic complications were observed in five patients (4.5%), including four hepatic ischemias and one bowel ischemia; two hepatic ischemias were directly related to MAL management. Overall postoperative mortality was 4.3%, with two deaths attributable to MAL-related hepatic ischemia.

**Conclusions:**

MAL is rare but clinically impactful in patients undergoing PD. Early recognition and appropriate intraoperative assessment are essential to prevent ischemic complications. When properly identified and managed, MAL does not appear to significantly worsen overall postoperative outcomes. Management should remain individualized, and pancreatic surgeons should be prepared to perform MAL division and vascular reconstruction when indicated.

**Supplementary Information:**

The online version contains supplementary material available at https://doi.org/10.1245/s10434-026-19830-8.

Coeliac trunk (CT) stenosis may arise from different etiologies, the most frequent being atherosclerosis and extrinsic compression by the median arcuate ligament (MAL). The presence of a median arcuate ligament (MAL) is observed in approximately 2–7% of patients undergoing pancreaticoduodenectomy (PD).^1,2^ The mechanism involves extrinsic compression of the coeliac trunk (CT) by a fibrous band, leading to partial or complete occlusion. Occlusion of the CT can result in reversed blood flow within the gastroduodenal artery (GDA), with the vascularization of the celiac territory, particularly the liver, becoming entirely dependent on the superior mesenteric artery.^3,4^

Preoperative (or intraoperative) diagnosis of MAL is crucial to prevent postoperative hepatic, splenic, or digestive ischemic complications. The gold standard diagnostic method is a contrast-enhanced computed tomography scan with arterial phase imaging.^5^ Intraoperatively, the GDA clamping test allows confirmation of the absence of hemodynamic impact by manual palpation of the hepatic artery pulse or by hepatic Doppler ultrasound.^6^

When a MAL with hemodynamic significance is present, several management options either radiological or surgical have been described in the literature. Preoperatively, endovascular dilation and stenting may be performed. Intraoperatively, two main approaches have been reported: division of the ligament or vascular bypass.^4–7^

The existing literature on this subject is notably limited, with most publications being isolated case studies and only a few series documented.^3,6–13^ At present, there is a scarcity of research assessing various management strategies, their associated morbidity and mortality rates, and existing studies are constrained by limited patient sample sizes.

While recent large multicenter studies have evaluated the global impact of celiac axis stenosis on outcomes after pancreatoduodenectomy, including the prognostic significance of stenosis severity and etiology, detailed data focusing specifically on management strategies, technical failures, and salvage procedures related to median arcuate ligament-associated compression remain scarce. In particular, how MAL is diagnosed, managed intraoperatively, and rescued in case of failure in real-life surgical practice has not been well characterized.

The objectives of this study are to describe the management options for MAL within the context of PD at expert centers and to evaluate postoperative outcomes on the basis of the chosen strategy.

## Patients and Methods

This is a retrospective multicenter study conducted with the support of the French Association of Hepato-Bilio-Pancreatic Surgery and Transplantation (ACHBPT). The study includes patients diagnosed with MAL regardless of the intraoperative hemodynamic impact, who underwent PD between 2005 and 2024 in ten high-volume pancreatic surgery centers. We defined a high-volume pancreatic surgery center as a center performing 20 or more pancreatic resection per year.^14^

All PD, regardless of the indication, were included. Patients younger than 18 years and those with a history of left pancreatectomy were excluded.

A practice survey was distributed across the participating centers affiliated with the ACHBPT (Supplement 1). One representative surgeon per center completed the survey according to their institutional practices. Data regarding patient baseline characteristics, preoperative imaging, and perioperative and postoperative course were collected in a retrospective database. Specific data on MAL included the presence and degree of stenosis, type of intraoperative exploration and treatment for each patient, as well as each center’s local policy in the setting of MAL.

A preoperative contrast-enhanced computed tomography scan of the abdomen with arterial phase is generally recommended.^9,15,16^ The MAL was defined as a fibrous arch formed by the union of the right and left diaphragmatic crura over the aortic hiatus, that causes stenosis of the CT through extrinsic compression.^12,17^ A partial MAL was defined as partial or incomplete stenosis of the lumen of the CT, whereas a total MAL was defined as complete stenosis of the lumen of the CT. All imaging studies were interpreted by either a qualified surgeon, radiologist, or both.

In this study, the term MAL refers to median arcuate ligament–related celiac axis compression. Cases with hemodynamic impact correspond to clinically significant MAL, whereas asymptomatic anatomical compression without hemodynamic consequence was considered nonsignificant.

According to the purpose of the study, a MAL with hemodynamic impact was considered in the following cases: complete occlusion of the CT related to the presence of a MAL on the preoperative CT scan with evidence of prominent collaterals, and positive GDA clamping test in the presence of MAL defined as a significant decrease or absence of hepatic artery pulse following intraoperative clamping of the GDA at manual palpation of the hepatic pedicle or Doppler ultrasound.

Treatment options considered for MAL were: preoperative endovascular stenting: placement of a stent in the CT prior to PD to treat fixed arterial stenosis and restore normal blood flow; MAL division also referred to as MAL release: surgical procedure consists of transecting the fibrous MAL and associated perivascular fibrotic tissue to relieve extrinsic compression of the CT and restore normal arterial blood flow during the same procedure as the PD; and vascular reconstruction refers to surgical restoration of adequate blood flow in the CT or its branches using techniques, such as arterial transposition, bypass grafting, or end-to-end/side-to-side anastomosis, during the same procedure as the PD.

Treatment failure of the MAL was defined as a positive clamping test after treatment and/or the occurrence of signs of hepatic, splenic, or digestive ischemia owing to persistent MAL. Postoperative period was defined as the first 90 days after PD. Postoperative morbidity was classified according to the Clavien–Dindo score.^18^ Mortality was defined as any death occurring within 90 days after surgery.

Race and ethnicity data were not included in this study because they are not routinely collected in the clinical records used for this analysis and were therefore unavailable.

Quantitative variables were reported as median (interquartile range), and qualitative variables as counts and percentages.

## Results

In total, 10 French centers participated in this study, including 115 patients who underwent a PD with suspected MAL stenosis among the 8676 PD performed during the study period.

### Systematic Management Strategy of MAL by Different Teams

A practice survey was distributed among surgeons from the different participating centers, comprising three questions (Supplement 1): 1. What intraoperative method is used to evaluate the presence of a MAL with hemodynamic impact: digital GDA clamping test or hepatic Doppler ultrasound? Seven centers systematically perform a GDA clamping test by manual palpation, and two centers add a hepatic Doppler ultrasound in addition to the clamping test. One center does not routinely perform the clamping test because they systematically cut the diaphragmatic crura. 2. What is the first-line treatment upon diagnosis of a MAL with hemodynamic impact? Three centers chose preoperative radiologic stent placement as first-line treatment for a MAL, while seven centers opted for ligament release during PD as the first-line treatment. 3. In which cases is vascular reconstruction justified in the presence of MAL? All centers agree that vascular reconstruction in the presence of MAL should be reserved after failure of ligament release or radiologic stenting, or when the MAL is too tight and division is not feasible.

### Baseline Characteristics

Baseline characteristics of the study population are presented in Table 1. The median age of patients was 68 years (61–75 years), and 58% (*n* = 67) were male. The majority of patients had an Eastern Clinical Oncology Group (ECOG) performance status of 0–1 (*n* = 95, 83%). Surgical indications for PD were tumor-related in > 90% of cases, with 60% of the patients (*n* = 69) diagnosed with pancreatic adenocarcinoma. In total, 30 patients (30%) received neoadjuvant chemotherapy, mainly Folfirinox (*n* = 26, 67%).

**Table 1:** Baseline characteristics

	*n*	%
Sex (M)	67	58
Age (years, median (IQR))	68.1 (61.0, 74.9)	
BMI (kg/m^2,^ median (IQR))	23.7 (20.1, 26.0)	
WHO		
0–1	95	83
2	20	17
Pathology		
Pancreas adenocarcinoma	69	60
NET	3	2.6
Cholangiocarcinoma	14	12
Ampulloma	15	13
Other cancers	6	5.2
Benign pathology	8	7
Neoadjuvant chemotherapy	30	27
Folfirinox	26	87
Neoadjuvant radiotherapy	8	7
Surgical approach: laparotomy	105	91
Operative duration (min, median (IQR))	380.0 (330–460)	
Blood loss (ml, median (IQR))	325.0 (200–600)	
Transfused patients	20	17
Pancreaticojejunostomy	94	82
Vascular resection unrelated to the MAL	36	31

Regarding the procedure, 91% of PD were carried out via laparotomy (*n* = 105), with a median operative time of just over 6 h (330–460 min) and estimated median blood loss of 325 cc (200–600 cc). Overall, 94 patients (82%) underwent a pancreaticojejunostomy and one-third (*n* = 36) had a vascular resection unrelated to the management of the MAL.

### Diagnosis of the MAL

A suspected MAL stenosis was identified in 115 patients (1.3%) among the 8676 PD performed during the study period across the 10 centers. In total, 5 cases (4.3%) were defined as total MAL and 110 cases (95.6%) partial MAL.

The preoperative assessment included contrast-enhanced computed tomography scans with arterial phase imaging in 110 patients (95.6%), while 5 patients (4.4%) had contrast-enhanced computed tomography scans without a specific arterial phase.

The diagnosis of MAL was suspected during the preoperative period in 104 patients (90%) and during the intraoperative period in 10 patients (9%). In one patient, the presence of a MAL was identified postoperatively following liver failure. In all patients without a preoperative diagnosis of MAL, a computed tomography scan with an arterial phase had been performed preoperatively, except for the patient who was diagnosed with MAL in the postoperative period, who had undergone a preoperative computed tomography scan without arterial contrast injection.

Among the 104 patients with preoperative diagnosis of MAL, 11 patients (9.5%) underwent complementary preoperative arteriography to confirm the suspicion of hemodynamically significant MAL, 8 of them were treated during the same procedure.

Overall, the rate of MAL presence with hemodynamic impact was 0.7% (*n* = 59) related to the presence of preoperative indirect radiological signs in 49 patients and/or intraoperative GDA clamping test in 46 patients.

### Treatment of the MAL

**Fig. 1:**
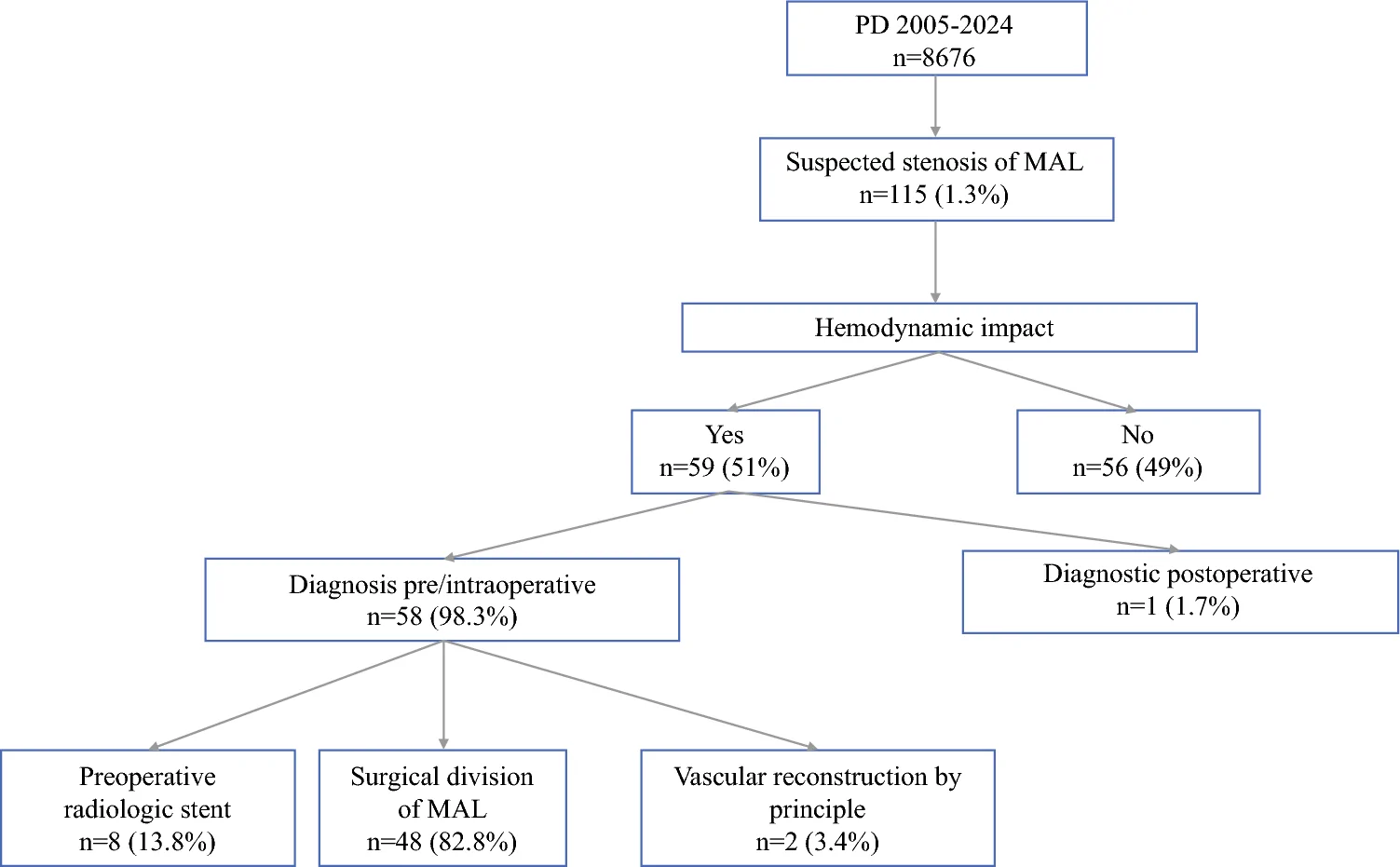
Management performed for MAL

Figure 1 shows the management performed. In total, 56 patients (49%) with suspected MAL stenosis did not receive further radiological exploration or specific treatment. In all these cases, intraoperative assessment confirmed the absence of hemodynamic impact, with preserved hepatic arterial flow after gastroduodenal artery clamping, leading to a deliberate decision not to perform MAL release or revascularization. These patients therefore represent confirmed cases of anatomically suspected but hemodynamically nonsignificant MAL. Patients diagnosed intraoperatively or postoperatively were included in the hemodynamically significant group.

In case of a MAL with hemodynamic impact, treatment involved preoperative radiological angiography for 8 patients (13.8%), intraoperative MAL division for 48 patients (82.8%), and vascular reconstruction as a primary strategy for 2 patients (3.4%) (Table 2). The success rate of these techniques was 100% (*n* = 8), 86% (*n* = 42), and 100% (*n* = 2), respectively.

**Table 2:** Treatment modality of the MAL

	*n*	%	Procedure failure
Specific treatment of the MAL			7 (12%)
Yes	58	50,4	
No	57	49,6	
Type of treatment			
Preoperative radiologic stent	8	7	0
Intraoperative MAL division	49	41.7	7 (14%)
In principle vascular reconstruction during PD	2	1.7	0
- Aorto-GDA bypass with cryopreserved graft	1	0.85	
- Spleno-hepatic anastomosis	1	0.85	

Failure of intraoperative MAL division occurred in seven patients (14%). In six cases (85%), failure was identified intraoperatively by persistent absence of adequate hepatic arterial flow following ligament release. Notably, all failures occurred in patients with partial celiac axis stenosis on preoperative computed tomography imaging. Salvage treatment was performed intraoperatively in five patients through vascular reconstruction, while one patient required postoperative endovascular balloon dilation. Only one failure was diagnosed postoperatively, in a patient who developed fatal hepatic ischemia despite vascular reconstruction on postoperative day (POD) 1. The timing of diagnosis, salvage strategies, and outcomes of these patients are detailed in Table 3.

**Table 3:** Salvage strategies implemented after failure of intraoperative median arcuate ligament (MAL) division

	Timing of failure	Salvage treatment	Interposed graft	MAL-specific morbidity
Patient 3: Aorto-hepatic bypass with saphenous vein graft	Intraoperative	Vascular reconstruction	Yes (saphenous)	No
Patient 5: Aorto-hepatic bypass with Gore-Tex graft	Intraoperative	Vascular reconstruction	Yes (Gore Tex©)	No
Patient 36: Dilatation of CT ostium with Fogarty catheter	Intraoperative	Endovascular balloon dilation	No	No
Patient 60: GDA–right gastroepiploic artery anastomosis	Intraoperative	Vascular reconstruction	No	No
Patient 83: GDA– pancreaticoduodenal artery anastomosis	Intraoperative	Vascular reconstruction	No	No
Patient 111: Hepatic artery–splenic artery anastomosis	Postoperative (POD1)	Vascular reconstruction	No	Hepatic ischemia. Death
Patient 115: GDA–right hepatic artery anastomosis	Intraoperative	Vascular reconstruction	No	No

### General Morbidity and Mortality

The median length of hospital stay was 16 days (11–21 days), with nine patients (8%) being readmitted during the postoperative period. Overall, 29 patients (25%) experienced a Clavien–Dindo complication of grade III or higher, with a rate of surgical reoperation of 8.7% (*n* = 10) and radiological intervention of 10.4% (*n* = 12). The rate of pancreatic fistula grade B–C was 10% (*n* = 12), and hemorrhage occurred in 15% (*n* = 17). In total, eight patients (6.9%) presented a biliary leakage treated with conservative management in all cases; none of these patients showed signs related to biliary ischemia owing to the treatment of MAL (Table 4).

**Table 4:** Morbidity and mortality at POD 90

	*n*	%
Length of hospital stay (days, median (IQR))	16.0 (11.0, 21.0)	
Clavien–Dindo ≥ III	29	25.2
Pancreatic fistula B–C	12	10.4
Biliary fistula	8	6.9
Hemorrhage	17	15
Reoperation	10	8.7
Death	5	4.3

In total, five patients (4.5%) developed ischemic complications (four hepatic ischemias and one bowel ischemia). One patient, with intraoperative diagnosis of MAL, experienced hepatic ischemia with a CT scan demonstrating the absence of hepatic arterial blood flow on POD 1 after MAL division during PD. He was treated with a vascular bypass between the hepatic artery and the aorta using the patient’s splenic artery on POD 1. A second patient was diagnosed with MAL on POD 3 and treated by radiologic stenting on POD 3. Both patients died on POD 7 and 12, respectively. The other cases of ischemia not related to MAL management involved one patient with thrombosis of a vascular reconstruction at the level of the hepatic artery, which had been resected owing to tumor infiltration; one patient with thrombosis of a mesenteric venous reconstruction performed because of tumor infiltration; and a case of right colon ischemia with no apparent cause identified, but requiring return to the operating room.

Other complications related to MAL treatment included an intraoperative injury to the CT, repaired with simple suture, and a superficial hematoma at the puncture site following placement of a preoperative radiologic stent. Specific complications related to MAL management are presented in Table 5.

**Table 5:** Specific morbidity related to the MAL

	Sex and age (years)	Pathology	Diagnosis of MAL	Treatment of MAL	Complication	Diagnosis of complication (POD)	Treatment	Postoperative course
Patient 7	F, 74	Pancreas ADK	Postoperative	No	Hepatic ischemia	3	Reoperation: diagnosis of MAL. Then radiologic stent (POD 3)	Death on POD 7
Patient 80	F, 61	Ampulloma	Preoperative	Preoperative radiologic stent	Hematoma at the puncture site	0	No specific treatment	Uncomplicated
Patient 86	F, 67	Pancreas ADK	Preoperative	Division of MAL	Millimetric injury to the CT	0	Simple suture (blood loss during surgery 500 cc)	Uncomplicated
Patient 111	F, 75	Pancreas ADK	Intraoperative	Division of MAL	Hepatic ischemia	1	Vascular reconstruction: hepatic artery–splenic artery anastomosis (POD 1)	Death on POD 12

Overall mortality rate was 4.3% (*n* = 5) owing to three hepatic ischemias (two related to MAL management), one hemorrhage from the pancreaticojejunal anastomosis, and one case of sepsis.

## Discussion

**Fig. 2:**
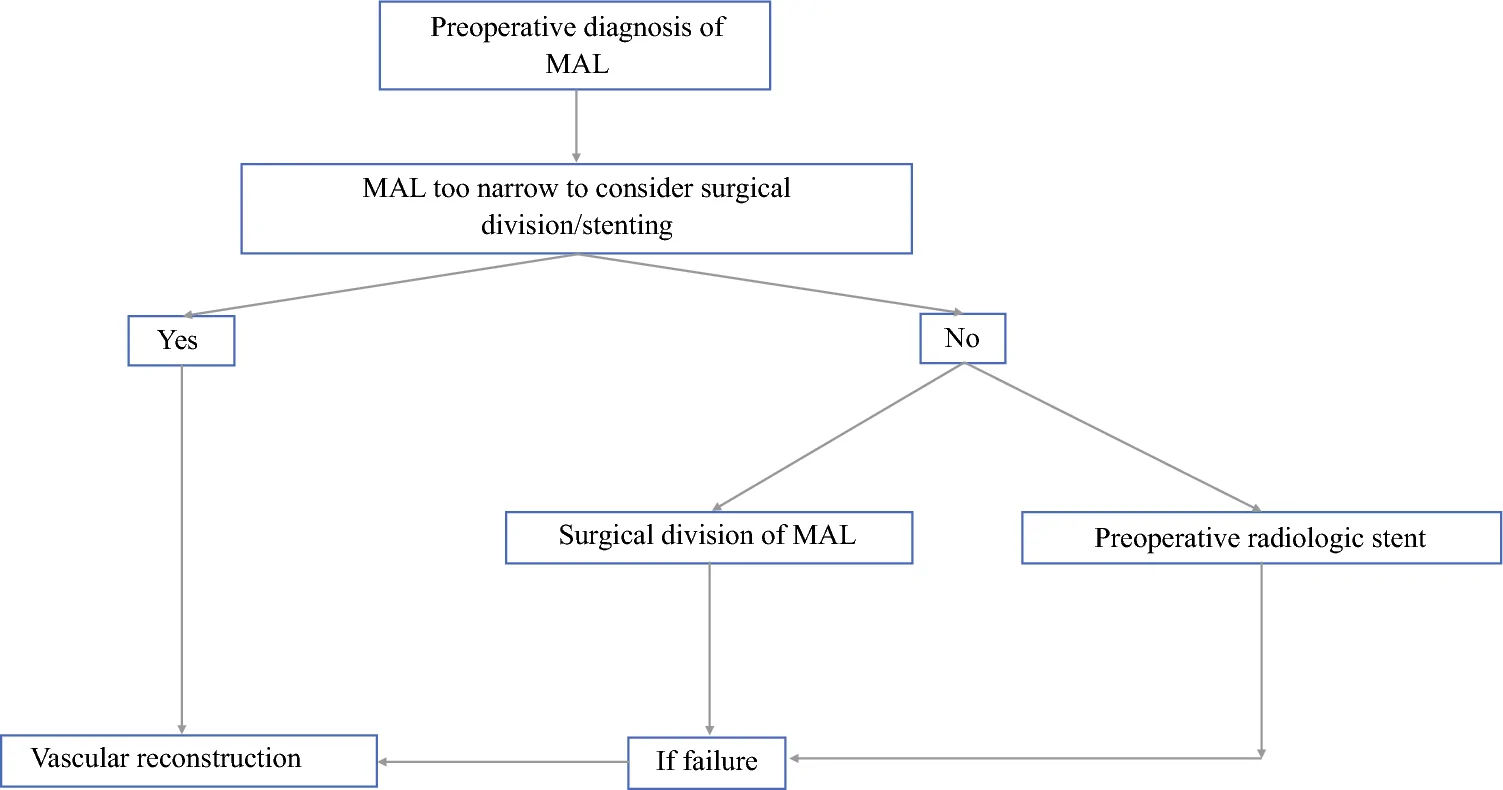
Proposed management algorithm for MAL, which represents a pragmatic management framework on the basis of current practices in high-volume centers and does not imply a standardized or prescriptive diagnostic pathway; intraoperative assessment of hemodynamic significance should be adapted to local expertise and available resources

In contrast to recent comparative studies addressing the prognostic impact of celiac axis stenosis during pancreatoduodenectomy,^13^ the present multicenter study focuses on the practical management of median arcuate ligament-related compression. Rather than evaluating MAL as a risk factor per se, our aim was to describe diagnostic pathways, intraoperative decision-making, treatment strategies, and failure patterns when MAL is encountered in high-volume pancreatic surgery centers (Fig. 2).

**Fig. 3:**
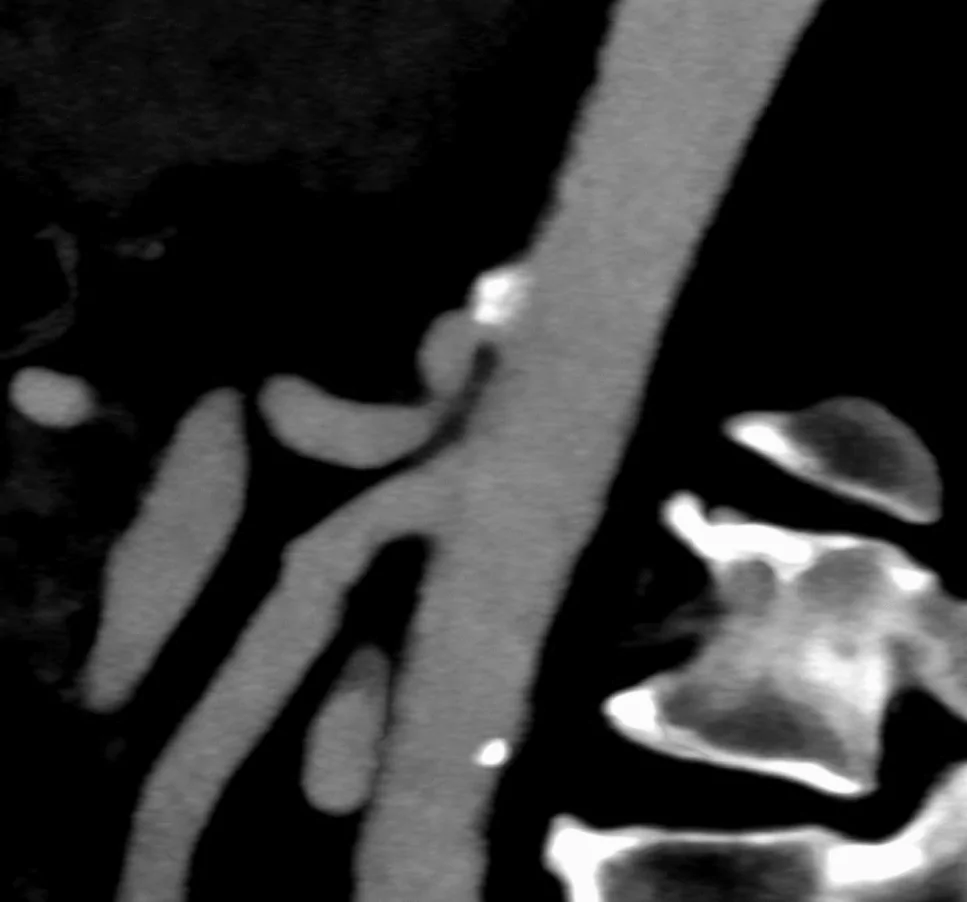
Calcified ostial atherosclerotic plaque without significant stenosis; severe, focal post-ostial stenosis due to MAL; photon-counting computed tomography scanner (Naeotom Alpha; Siemens Healthineers); ultra-high-resolution mode (0.4 mm)

The incidence of MAL in the general population is estimated between 2% and 7%, but its clinical significance varies widely.^1,2,9,13^ In most cases, it remains asymptomatic owing to the extensive collateral circulation between the superior mesenteric artery and the celiac territory. However, in patients undergoing PD, interruption of these collaterals by ligation of the GDA can unmask a previously silent MAL, leading to potentially catastrophic hepatic, gastric, or splenic ischemia.^3,9,11,19^ Within our cohort, stenosis caused by the MAL was detected in approximately 1.3% of patients undergoing PD. Of these cases, 0.7% exhibited compression that was deemed hemodynamically significant. This incidence is consistent with rates reported in previous surgical series, which range from 0.4% to 4%.^3,4,6,17,20^ The actual rate of nonhemodynamically significant stenosis may be underestimated because preoperative imaging is not always reviewed for this diagnosis. This highlights the relatively low but noteworthy frequency of MAL involvement in patients undergoing PD and emphasizes the importance of careful evaluation for MAL compression, particularly when hemodynamic significance is suspected. Early and accurate diagnosis of MAL is therefore crucial. Preoperative detection allows appropriate pre- or intraoperative strategy and anticipates salvage procedures (i.e., vascular reconstruction) to prevent postoperative ischemic risk.^6,7,13,17,21^ Contrast-enhanced computed tomography scan with arterial phase reviewed by experienced radiologist and surgeon remains the modality of choice for diagnosis. Imaging features of CT compression by MAL with hemodynamic impact include focal narrowing of the superior aspect of the proximal CT forming a hooked or “J” appearance associated with a post stenotic dilatation or evidence of prominent collaterals, such as the GDA and common hepatic artery (Fig. 3). Computed tomography scan is typically performed during inspiration because descent of the diaphragm results in caudal displacement of the MAL, and compression of the CT becomes more horizontal, with reduction or disappearance of the proximal narrowing. Persistence of stenosis during inspiration suggests significant compression.^9,15,16^ A standardized classification of celiac axis stenosis owing to MAL compression has not previously been established. In the last years, several authors suggested dedicated definition to MAL. Sugae et al. first proposed a radiological classification for patients undergoing PD according to preoperative imaging findings. This represented the first attempt to categorize the degrees of MAL compression,^7^ although this classification does not perfectly reflect reality, particularly regarding the relationship between CT stenosis and the development of collateral circulation. In this sense, Xu et al. showed that both the degree of CT stenosis and the extent of collateral circulation were significant indicators of symptomatic MAL.^22^ Despite their limitations, these classifications may help predict complications in the setting of MAL, as recently shown in a multicenter series.^13^ The role of diagnostic arteriography is limited and rarely performed in current practice owing to its invasive nature and the high diagnostic yield of computed tomography scans.^20^ Compared with arteriography, computed tomography provides a more convenient way to evaluate the collateral circulation with a lower dose exposure.^22^ In our cohort, very few patients underwent preoperative arteriography, which reflects current practice patterns in most high-volume centers. This reinforces the notion that high quality computed tomography provides sufficient anatomical and hemodynamic information in almost all cases. However, performing arteriography when a hemodynamically significant MAL is suspected could be useful, particularly if a concomitant stenting is considered as a therapeutic strategy.

When MAL is suspected or identified preoperatively, intraoperative confirmation of its hemodynamic impact is essential. The GDA clamping test, performed either by digital palpation or under Doppler ultrasound guidance, remains the most practical intraoperative evaluation.^13,23^ In our study, almost 80% of centers routinely performed manual clamping, while only a minority used intraoperative Doppler. Similar trends are described in other reports, underlining that Doppler assessment, although more objective, is not universally performed. Nevertheless, this test remains inherently subjective and heavily dependent on the surgeon’s experience. This variability contributes to the lack of standardized intraoperative criteria across institutions.^2,8,20,24^ Despite the inherent subjectivity of the procedure, our experience shows that diagnoses are generally made accurately in high-volume centers with only one case of misdiagnosis.

Several therapeutic strategies have been proposed in the literature.^2,3,5–7,12,13,25,26^ Guastella et al. have shown that centers with a standardized diagnostic assessment protocol performed treatments more frequently than those without.^13^ Preoperative endovascular stenting offers the advantage of immediate and controlled revascularization, avoiding intraoperative uncertainty. Small case series have shown this approach to be safe and effective.^3,13,21,27,28^ However, the need for dual antiplatelet therapy, the risk of in-stent thrombosis, the potential technical challenges in cases of severe stenosis, and logistical constraints (including availability of interventional radiologists and scheduling delays for oncologic patients) limit its widespread use. In our series, only 14% of treated patients underwent preoperative stenting, with a 100% success rate, in line with previous studies.^3,21^ Among the eight patients treated with preoperative radiological stenting, only one patient required intraoperative transfusion and no postoperative hemorrhagic complications were observed. Therefore, our series does not appear to demonstrate an increased risk of bleeding-related complications in patients undergoing preoperative stenting. Intraoperative division of MAL was the most frequently applied strategy in this multicenter cohort, reflecting current practice patterns in participating centers rather than comparative effectiveness, and accounted for the initial management in 82% of cases with hemodynamically significant MAL. However, this option is associated with a 14% failure rate requiring salvage vascular reconstructions. These results are comparable to those reported in the literature^6,19,23,26,27,29,30^ ranging between 2% and 14%. Failure after MAL division may occur owing to long-standing fibrotic changes or associated atherosclerotic disease.^30^ Vascular reconstruction, either by arterial transposition, bypass, or anastomosis, is generally indicated for cases of persistent ischemia after division.^6,24^ In some rare cases of severe MAL with complete occlusion this strategy can be planned as an up-front strategy. In our study, it was required as a primary strategy in only two cases and in six cases following intraoperative diagnosis of a failed release. These results align with previous reports, which emphasize that reconstruction should remain a last resort owing to its technical complexity and potential morbidity.^3,27^ Some reports propose preserving collateral branches as an additional technique in certain benign cases.^2,5^ However, the high rate of PD performed for malignant disease in our study likely accounts for the absence of this option. Although the limited number of failures precludes identification of formal predictors, the descriptive analysis of these cases provides pragmatic insights for intraoperative decision-making. Persistent absence of hepatic arterial flow after ligament release should be considered a critical warning sign, prompting immediate consideration of vascular or endovascular reconstruction. Interestingly, all failures of MAL division in our cohort occurred in patients with partial, rather than complete, celiac axis stenosis, underscoring that anatomical severity alone does not reliably predict hemodynamic impact or technical success. These observations should be interpreted as hypothesis-generating rather than predictive and reinforce the importance of meticulous intraoperative assessment and preparedness for salvage revascularization.

To our knowledge, this series represents the largest multicenter cohort reported to date on this topic. Overall morbidity and mortality in our study (25% ≥ Clavien–Dindo grade III, 4.3% mortality) were comparable to those reported for standard PD in high-volume centers.^31,32^ Importantly, ischemic complications occurred in only 4.5% of patients, with just two cases directly attributable to MAL management, both resulting in fatal hepatic ischemia. This incidence is slightly lower than the 5–10% rate of ischemic complications reported in previous smaller series, which emphasized the benefit of systematic preoperative or intraoperative assessment.^6,13,20,30^ When compared with the recently reported multicenter series by Guastella et al.,^13^ our data suggest that the mere presence of a MAL does not confer an increased risk of postoperative complications; rather, complications appear to be driven by unrecognized and therefore inadequately managed MAL. Although Al Saeedi et al. reported an association between hemodynamically significant MAL and the occurrence of grade B–C pancreatic fistula and biliary fistula, the incidence of these complications was not increased in our cohort. Taken together, these findings reinforce that the presence of a MAL, when properly diagnosed and managed, does not significantly worsen overall outcomes after PD.^19,33^

Beyond descriptive outcomes, our findings support a stepwise and pragmatic strategy for the management of MAL in patients undergoing PD. Accordingly, the proposed management framework should be interpreted as a pragmatic synthesis of fundamental decision-making principles rather than a time-specific pathway. This approach emphasizes systematic preoperative recognition, intraoperative confirmation of hemodynamic significance, MAL division as the most commonly applied initial strategy in this cohort, and prompt vascular reconstruction in cases of persistent ischemia or treatment failure. The key message from our experience is the paramount importance of preoperative diagnosis. Recognition of MAL before surgery allows optimal planning—whether by radiologic stenting or by anticipating intraoperative release—to prevent catastrophic hepatic or digestive ischemia. If preoperative detection is missed, intraoperative identification remains feasible through meticulous evaluation of hepatic arterial flow before the division of the GDA. However, a postoperative diagnosis almost invariably leads to severe complications and unfavorable outcomes. Pancreatic surgeons should remain aware of the critical importance of identifying significant MAL preoperatively, or at the latest intraoperatively, prior to formal resection, and should also be adequately trained in the technique of MAL division and vascular reconstructions to prevent potentially fatal complications. On the basis of these findings, we propose the following pragmatic management algorithm to support decision-making when MAL is encountered during pancreatoduodenectomy (Fig. 2).

This study has several strengths, including the largest multicenter cohort reported to date on this topic, standardized data collection, and a pragmatic depiction of real-world surgical practice, allowing the proposal of a management framework for MAL encountered during pancreatoduodenectomy. Nevertheless, inherent limitations must be acknowledged. The retrospective design and heterogeneity of practices across centers reflect the absence of consensus-based recommendations and limit cross-center comparability. In addition, the extended study period introduces potential temporal heterogeneity related to evolving imaging quality, diagnostic awareness, endovascular techniques, and perioperative management, which may affect cross-era comparability. In particular, the lack of uniform criteria for defining hemodynamically significant MAL—given the variability in intraoperative assessment using manual GDA clamping, Doppler ultrasound, or systematic crural division—may have influenced patient classification. Consequently, any proposed management strategy must remain adaptable and guided by local expertise rather than relying on a single diagnostic modality. Furthermore, owing to the small and markedly unbalanced number of patients across management strategies, no formal comparison between preoperative stenting, MAL division, and vascular reconstruction could be undertaken, and no conclusions regarding the superiority or comparative effectiveness of any specific approach can be drawn.

In conclusion, MAL is rare but clinically impactful in patients undergoing pancreaticoduodenectomy. Early identification, ideally during the preoperative workup, is essential to prevent potentially life-threatening ischemic complications. In this multicenter experience, surgical division of the ligament was the most commonly applied initial strategy, while vascular reconstruction was required in selected cases of persistent ischemia or treatment failure. Given the descriptive nature of the study and the absence of comparative data, management decisions should remain individualized and guided by anatomical findings, intraoperative hemodynamic assessment, and institutional expertise. Pancreatic surgeons should be familiar with MAL division and prepared for vascular reconstruction to minimize ischemic risk.

## Supplementary Information

Below is the link to the electronic supplementary material.

Supplementary file1 (DOCX 13 KB)
