# Supplementary material for: How to Deal with Median Arcuate Ligament during Pancreaticoduodenectomy? Management Strategies and Impact on Perioperative Outcomes
Source: Ann Surg Oncol. 2026 May 23;33(12):10090–9. doi: 10.1245/s10434-026-19830-8 (PMC13675262; doi:10.1245/s10434-026-19830-8)
Supplement: Supplementary file 1 [file 10434_2026_19830_MOESM1_ESM.docx]

**Supplement 1: Practice survey distributed among surgeons from different participations centers**

1. **What intraoperative method is used to evaluate the presence of an MAL with hemodynamic impact: digital GDA clamping test or hepatic Doppler ultrasound?**
   a) **GDA clamping test by manual palpation**
   b) **Hepatic Doppler ultrasound in addition to the clamping test by manual palpation**
   c) **Other (Could you describe it, please?)**
2. **What is the first-line treatment upon diagnosis of a MAL with hemodynamic impact?**
   a) **Preoperative radiologic stent placement**
   b) **MAL division**
   c) **Vascular bypass**
   d) **Other (Could you describe it, please?)**
3. **In which cases is vascular bypass justified in the presence of an MAL?**
   a) **First line of treatment**
   b) **After failure of MAL division/ radiologic stent**
   c) **MAL too tight and division is not feasible**
   d) **Other (Could you describe it, please?)**
